# 2-Acetyl-1,1,3,3-tetra­methyl­guanidine

**DOI:** 10.1107/S1600536812039724

**Published:** 2012-09-26

**Authors:** Ioannis Tiritiris

**Affiliations:** a Fakultät Chemie/Organische Chemie, Hochschule Aalen, Beethovenstrasse 1, D-73430 Aalen, Germany

## Abstract

In the mol­ecule of the title compound, C_7_H_15_N_3_O, the central C atom is surrounded in a nearly ideal trigonal–planar geometry by three N atoms. The C—N bond lengths in the CN_3_ unit are 1.3353 (13), 1.3463 (12) and 1.3541 (13) Å, indicating an inter­mediate character between a single and a double bond for each C—N bond. The bonds between the N atoms and the terminal C-methyl groups all have values close to that of a typical single bond [1.4526 (13)–1.4614 (14) Å]. In the crystal, the guanidine mol­ecules are connected by weak C—H⋯O and C—H⋯N hydrogen bonds, generating layers parallel to the *ab* plane.

## Related literature

For the preparation of *N*-acetyl-*N*′,*N*′,*N*′′,*N*′′-tetra­methyl­guanidine, see: Kessler & Leibfritz (1970 ▶). For the preparation and properties of acyl­guanidines, see: Matsumoto & Rapoport (1968 ▶).
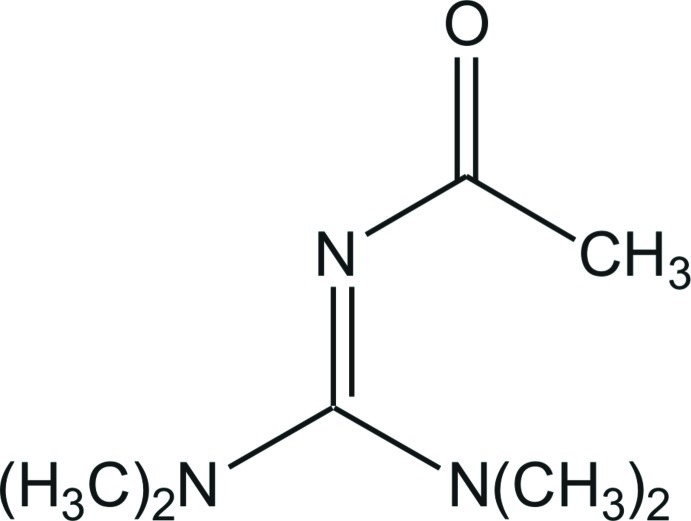


## Experimental

### 

#### Crystal data

C_7_H_15_N_3_O
*M* *_r_* = 157.22
Monoclinic, 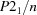

*a* = 6.7625 (3) Å
*b* = 17.8610 (8) Å
*c* = 7.6687 (4) Å
β = 103.107 (2)°
*V* = 902.13 (7) Å^3^
*Z* = 4
Mo *K*α radiation
μ = 0.08 mm^−1^
*T* = 100 K
0.22 × 0.18 × 0.16 mm

#### Data collection

Bruker Kappa APEXII Duo diffractometer
17552 measured reflections
2758 independent reflections
2396 reflections with *I* > 2σ(*I*)
*R*_int_ = 0.031

#### Refinement

*R*[*F* ^2^ > 2σ(*F* ^2^)] = 0.046
*wR*(*F* ^2^) = 0.117
*S* = 1.10
2758 reflections
105 parameters
H-atom parameters constrained
Δρ_max_ = 0.29 e Å^−3^
Δρ_min_ = −0.22 e Å^−3^

### 

Data collection: *APEX2* (Bruker, 2008 ▶); cell refinement: *SAINT* (Bruker, 2008 ▶); data reduction: *SAINT*; program(s) used to solve structure: *SHELXS97* (Sheldrick, 2008 ▶); program(s) used to refine structure: *SHELXL97* (Sheldrick, 2008 ▶); molecular graphics: *DIAMOND* (Brandenburg & Putz, 2005 ▶); software used to prepare material for publication: *SHELXL97*.

## Supplementary Material

Crystal structure: contains datablock(s) I, global. DOI: 10.1107/S1600536812039724/zl2504sup1.cif

Structure factors: contains datablock(s) I. DOI: 10.1107/S1600536812039724/zl2504Isup2.hkl

Supplementary material file. DOI: 10.1107/S1600536812039724/zl2504Isup3.cml

Additional supplementary materials:  crystallographic information; 3D view; checkCIF report

## Figures and Tables

**Table 1:** Hydrogen-bond geometry (Å, °)

*D*—H⋯*A*	*D*—H	H⋯*A*	*D*⋯*A*	*D*—H⋯*A*
C3—H3*B*⋯O1^i^	0.98	2.60	3.4807 (10)	150
C5—H5*A*⋯N3^ii^	0.98	2.61	3.5456 (15)	160

Symmetry codes: (i) 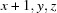; (ii) 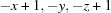.

**Fig. 1.**
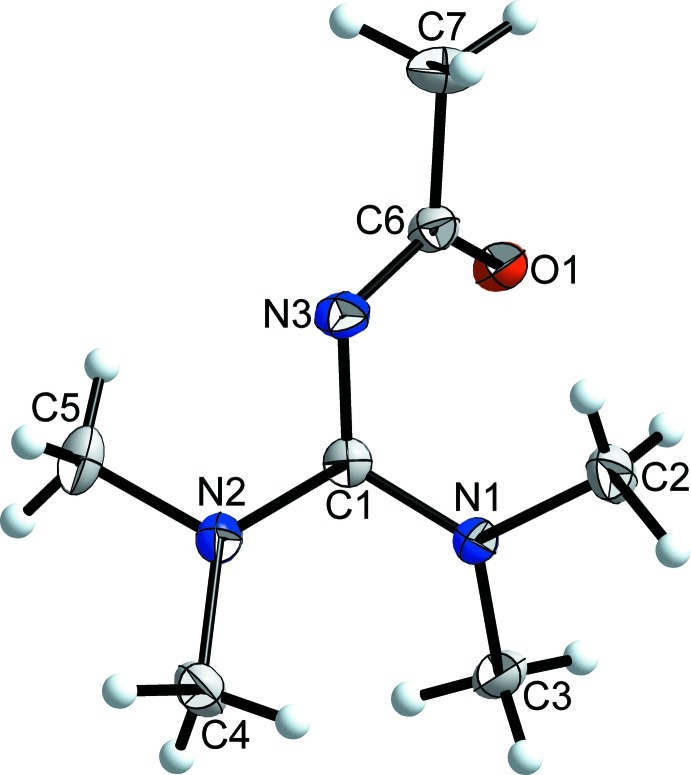
The structure of the title compound with atom labels and 50% probability displacement ellipsoids.

**Fig. 2.**
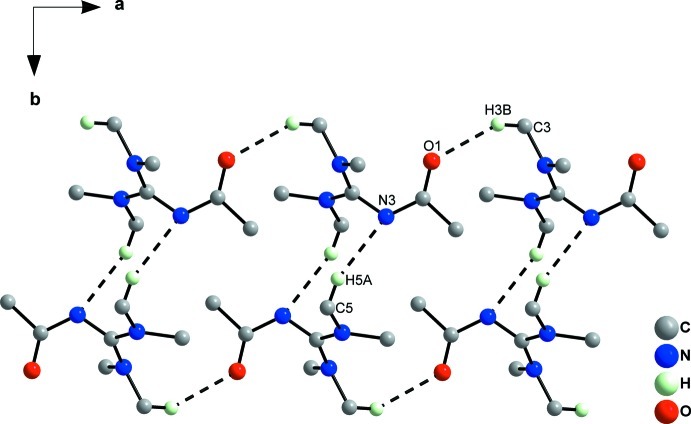
C—H···O and C—H···N hydrogen bonds between the guanidine molecules, ab view. The hydrogen bonds are indicated by dashed lines.

## supplementary crystallographic information

### Comment

The preparation and properties of various acylguanidines have been described in the literature several years ago (Matsumoto & Rapoport, 1968). While in acylguanidines it can be distinguished between the acylamino and and acylimino form, the increase in pK_a_ going from acylimino- to acylaminoguanidines was explained by conjugation of the guanidine part with the acetyl group. The here presented acylimino type title compound was described in the literature as a colorless liquid (Kessler & Leibfritz, 1970), but quite recently it was possible to obtain single crystals and to elucidate its hitherto unknown crystal structure. According to the structure analysis, the C—N bond lengths of the CN_3_ unit are: C1—N3 = 1.3353 (13) Å, C1—N2 = 1.3463 (12) Å and C1—N1 = 1.3541 (13) Å. They appear intermediate between those expected for single and double C—N bonds (1.46 and 1.28 Å, respectively). The N—C1—N angles are: 117.99 (9)° (N1—C1—N2), 118.62 (9)° (N2—C1—N3) and 123.11 (9)° (N1—C1—N3), which indicates only a slight deviation from an ideal trigonal–planar surrounding of the carbon centre by the N atoms. The bonds between the N atoms and the terminal C-methyl groups all have values close to a typical single bond (1.4526 (13)–1.4614 (14) Å). The bond lengths in the acetyl group are: C6—O1 = 1.2325 (13) Å, C6—C7 = 1.5109 (15) Å and N3—C6 = 1.3554 (13) Å. The C—O bond shows the expected double-bond character while the C—C bond is typical for a single bond. The dihedral angle C1—N3—C6—C7 is -166.23 (10)° and the angle between the planes N1/C1/N2 and C7/C6/O1 is 58.49 (10)°, which shows a significant twisting of the acetyl group relative to the CN_3_ plane (Fig. 1). Only weak C—H···O and C—H···N hydrogen bonds between methyl H atoms and carbonyl O atoms or N atoms of neighboring acetylguanidine molecules have been determined [*d*(H···O) = 2.60 Å and *d*(H···N) = 2.61 Å] (Table 1), generating chains along (100) (Fig. 2).

### Experimental

The title compound was obtained by heating two equivalents of *N*',*N*',*N*'',*N*''-tetramethylguanidine with one equivalent acetyl chloride in acetonitrile for 2 h under reflux (Kessler & Leibfritz, 1970). After cooling at room temperature the precipitated *N*',*N*',*N*'',*N*''-tetramethylguanidinium chloride was filtered off and the solvent was removed. The residue was redissolved in diethylether and the insoluble part was filtered off. After evaporation of the solvent a colorless liquid has been obtained. The title compound crystallized spontaneously after several days during standing at room temperature, giving colorless single crystals suitable for X-ray analysis. ^1^H NMR (500 MHz, CDCl_3_/TMS): δ = 2.10 (s, 3 H, CH_3_), 2.90 [s, 12 H, N(CH_3_)_2_]. ^13^C NMR (125 MHz, CDCl_3_/TMS): δ = 26.3 (CH_3_), 40.0 [N(CH_3_)_2_], 166.7 (C═N), 178.8 (C═O).

### Refinement

The H atoms of the methyl groups were allowed to rotate with a fixed angle around the C—N or C—C bond to best fit the experimental electron density, with *U*(H) set to 1.5 *U*_eq_(C) and d(C—H) = 0.98 Å.

### Crystal data

**Table:**

C_7_H_15_N_3_O	*F*(000) = 344
*M**_r_* = 157.22	*D*_x_ = 1.158 Mg m^−^^3^
Monoclinic, *P*2_1_/*n*	Mo *K*α radiation, λ = 0.71073 Å
Hall symbol: -P 2yn	Cell parameters from 17552 reflections
*a* = 6.7625 (3) Å	θ = 2.3–30.6°
*b* = 17.8610 (8) Å	µ = 0.08 mm^−^^1^
*c* = 7.6687 (4) Å	*T* = 100 K
β = 103.107 (2)°	Block, colourless
*V* = 902.13 (7) Å^3^	0.22 × 0.18 × 0.16 mm
*Z* = 4	

### Data collection

**Table:**

Bruker Kappa APEXII Duo diffractometer	2396 reflections with *I* > 2σ(*I*)
Radiation source: sealed tube	*R*_int_ = 0.031
Graphite monochromator	θ_max_ = 30.6°, θ_min_ = 2.3°
φ scans, and ω scans	*h* = −9→9
17552 measured reflections	*k* = −25→25
2758 independent reflections	*l* = −10→10

### Refinement

**Table:**

Refinement on *F*^2^	Primary atom site location: structure-invariant direct methods
Least-squares matrix: full	Secondary atom site location: difference Fourier map
*R*[*F*^2^ > 2σ(*F*^2^)] = 0.046	Hydrogen site location: difference Fourier map
*wR*(*F*^2^) = 0.117	H-atom parameters constrained
*S* = 1.10	*w* = 1/[σ^2^(*F*_o_^2^) + (0.0482*P*)^2^ + 0.2933*P*] where *P* = (*F*_o_^2^ + 2*F*_c_^2^)/3
2758 reflections	(Δ/σ)_max_ < 0.001
105 parameters	Δρ_max_ = 0.29 e Å^−^^3^
0 restraints	Δρ_min_ = −0.22 e Å^−^^3^

### Special details

**Table:**

Geometry. All e.s.d.'s (except the e.s.d. in the dihedral angle between two l.s. planes) are estimated using the full covariance matrix. The cell e.s.d.'s are taken into account individually in the estimation of e.s.d.'s in distances, angles and torsion angles; correlations between e.s.d.'s in cell parameters are only used when they are defined by crystal symmetry. An approximate (isotropic) treatment of cell e.s.d.'s is used for estimating e.s.d.'s involving l.s. planes.
Refinement. Refinement of *F*^2^ against ALL reflections. The weighted *R*-factor *wR* and goodness of fit *S* are based on *F*^2^, conventional *R*-factors *R* are based on *F*, with *F* set to zero for negative *F*^2^. The threshold expression of *F*^2^ > σ(*F*^2^) is used only for calculating *R*-factors(gt) *etc*. and is not relevant to the choice of reflections for refinement. *R*-factors based on *F*^2^ are statistically about twice as large as those based on *F*, and *R*-factors based on ALL data will be even larger.

### Fractional atomic coordinates and isotropic or equivalent isotropic displacement parameters (Å^2^)

**Table:**

	*x*	*y*	*z*	*U*_iso_*/*U*_eq_	
C1	0.47479 (14)	0.12793 (5)	0.62624 (13)	0.01806 (19)	
N1	0.59364 (13)	0.17507 (5)	0.74330 (11)	0.02142 (19)	
N2	0.51335 (13)	0.12040 (5)	0.46244 (11)	0.02139 (19)	
N3	0.33436 (14)	0.08392 (5)	0.67072 (13)	0.02306 (19)	
C2	0.60794 (18)	0.16825 (8)	0.93451 (14)	0.0306 (3)	
H2A	0.5062	0.2006	0.9691	0.046*	
H2B	0.7439	0.1834	0.9999	0.046*	
H2C	0.5834	0.1161	0.9636	0.046*	
C3	0.66323 (17)	0.24704 (6)	0.69034 (16)	0.0266 (2)	
H3A	0.6170	0.2532	0.5605	0.040*	
H3B	0.8119	0.2488	0.7236	0.040*	
H3C	0.6076	0.2874	0.7513	0.040*	
C4	0.71272 (17)	0.13280 (7)	0.42383 (15)	0.0281 (2)	
H4A	0.8119	0.1437	0.5355	0.042*	
H4B	0.7054	0.1752	0.3415	0.042*	
H4C	0.7546	0.0878	0.3686	0.042*	
C5	0.37019 (18)	0.08048 (6)	0.32279 (15)	0.0284 (2)	
H5A	0.4177	0.0290	0.3145	0.043*	
H5B	0.3601	0.1059	0.2079	0.043*	
H5C	0.2364	0.0796	0.3520	0.043*	
C6	0.19940 (15)	0.11382 (6)	0.75754 (14)	0.0223 (2)	
O1	0.15957 (12)	0.18080 (5)	0.76760 (12)	0.0296 (2)	
C7	0.08363 (19)	0.05584 (8)	0.83782 (18)	0.0348 (3)	
H7A	0.0030	0.0808	0.9120	0.052*	
H7B	0.1796	0.0211	0.9119	0.052*	
H7C	−0.0067	0.0280	0.7416	0.052*	

### Atomic displacement parameters (Å^2^)

**Table:**

	*U*^11^	*U*^22^	*U*^33^	*U*^12^	*U*^13^	*U*^23^
C1	0.0184 (4)	0.0164 (4)	0.0188 (4)	0.0027 (3)	0.0031 (3)	0.0017 (3)
N1	0.0197 (4)	0.0264 (4)	0.0180 (4)	−0.0044 (3)	0.0039 (3)	−0.0012 (3)
N2	0.0224 (4)	0.0225 (4)	0.0187 (4)	−0.0006 (3)	0.0036 (3)	−0.0016 (3)
N3	0.0244 (4)	0.0172 (4)	0.0293 (4)	−0.0019 (3)	0.0096 (3)	0.0001 (3)
C2	0.0254 (5)	0.0473 (7)	0.0180 (5)	−0.0033 (5)	0.0027 (4)	−0.0017 (4)
C3	0.0230 (5)	0.0250 (5)	0.0313 (5)	−0.0066 (4)	0.0053 (4)	−0.0039 (4)
C4	0.0263 (5)	0.0373 (6)	0.0229 (5)	0.0019 (4)	0.0102 (4)	−0.0010 (4)
C5	0.0340 (6)	0.0226 (5)	0.0245 (5)	0.0009 (4)	−0.0016 (4)	−0.0064 (4)
C6	0.0183 (4)	0.0243 (5)	0.0240 (5)	−0.0032 (3)	0.0043 (4)	−0.0002 (4)
O1	0.0233 (4)	0.0252 (4)	0.0408 (5)	0.0008 (3)	0.0086 (3)	−0.0054 (3)
C7	0.0309 (6)	0.0358 (6)	0.0410 (6)	−0.0085 (5)	0.0151 (5)	0.0053 (5)

### Geometric parameters (Å, º)

**Table:**

C1—N3	1.3353 (13)	C3—H3C	0.9800
C1—N2	1.3463 (12)	C4—H4A	0.9800
C1—N1	1.3541 (13)	C4—H4B	0.9800
N1—C2	1.4526 (13)	C4—H4C	0.9800
N1—C3	1.4579 (14)	C5—H5A	0.9800
N2—C5	1.4568 (13)	C5—H5B	0.9800
N2—C4	1.4614 (14)	C5—H5C	0.9800
N3—C6	1.3554 (13)	C6—O1	1.2325 (13)
C2—H2A	0.9800	C6—C7	1.5109 (15)
C2—H2B	0.9800	C7—H7A	0.9800
C2—H2C	0.9800	C7—H7B	0.9800
C3—H3A	0.9800	C7—H7C	0.9800
C3—H3B	0.9800		
			
N3—C1—N2	118.62 (9)	N2—C4—H4A	109.5
N3—C1—N1	123.11 (9)	N2—C4—H4B	109.5
N2—C1—N1	117.99 (9)	H4A—C4—H4B	109.5
C1—N1—C2	120.71 (9)	N2—C4—H4C	109.5
C1—N1—C3	122.97 (8)	H4A—C4—H4C	109.5
C2—N1—C3	113.79 (9)	H4B—C4—H4C	109.5
C1—N2—C5	119.86 (9)	N2—C5—H5A	109.5
C1—N2—C4	123.83 (9)	N2—C5—H5B	109.5
C5—N2—C4	114.53 (9)	H5A—C5—H5B	109.5
C1—N3—C6	119.38 (9)	N2—C5—H5C	109.5
N1—C2—H2A	109.5	H5A—C5—H5C	109.5
N1—C2—H2B	109.5	H5B—C5—H5C	109.5
H2A—C2—H2B	109.5	O1—C6—N3	126.46 (10)
N1—C2—H2C	109.5	O1—C6—C7	119.93 (10)
H2A—C2—H2C	109.5	N3—C6—C7	113.51 (10)
H2B—C2—H2C	109.5	C6—C7—H7A	109.5
N1—C3—H3A	109.5	C6—C7—H7B	109.5
N1—C3—H3B	109.5	H7A—C7—H7B	109.5
H3A—C3—H3B	109.5	C6—C7—H7C	109.5
N1—C3—H3C	109.5	H7A—C7—H7C	109.5
H3A—C3—H3C	109.5	H7B—C7—H7C	109.5
H3B—C3—H3C	109.5		
			
N3—C1—N1—C2	14.29 (15)	N3—C1—N2—C4	−147.74 (10)
N2—C1—N1—C2	−159.51 (10)	N1—C1—N2—C4	26.35 (15)
N3—C1—N1—C3	−146.52 (10)	N2—C1—N3—C6	−136.75 (10)
N2—C1—N1—C3	39.68 (14)	N1—C1—N3—C6	49.48 (14)
N3—C1—N2—C5	16.15 (14)	C1—N3—C6—O1	17.39 (17)
N1—C1—N2—C5	−169.76 (9)	C1—N3—C6—C7	−166.23 (10)

### Hydrogen-bond geometry (Å, º)

**Table:**

*D*—H···*A*	*D*—H	H···*A*	*D*···*A*	*D*—H···*A*
C3—H3*B*···O1^i^	0.98	2.60	3.4807 (10)	150
C5—H5*A*···N3^ii^	0.98	2.61	3.5456 (15)	160

Symmetry codes: (i) *x*+1, *y*, *z*; (ii) −*x*+1, −*y*, −*z*+1.
